# The Mitochondrial-Derived Peptide MOTS-c Alleviates Radiation Pneumonitis via an Nrf2-Dependent Mechanism

**DOI:** 10.3390/antiox13050613

**Published:** 2024-05-17

**Authors:** Yanli Zhang, Jianfeng Huang, Yaru Zhang, Fengjuan Jiang, Shengpeng Li, Shuai He, Jiaojiao Sun, Dan Chen, Ying Tong, Qingfeng Pang, Yaxian Wu

**Affiliations:** 1 Wuxi School of Medicine, Jiangnan University, 1800 Lihu Avenue, Wuxi 214122, China; zhangyl@stu.jiangnan.edu.cn (Y.Z.); zyr002@stu.jiangnan.edu.cn (Y.Z.); jiangfj2020@stu.jiangnan.edu.cn (F.J.); 7222808016@stu.jiangnan.edu.cn (S.L.); heshuai@stu.jiangnan.edu.cn (S.H.); 6202807023@stu.jiangnan.edu.cn (J.S.); 8201810174@jiangnan.edu.cn (D.C.); tongyhg@jiangnan.edu.cn (Y.T.); qfpang@jiangnan.edu.cn (Q.P.); 2 Affiliated Hospital of Jiangnan University, 1000 Hefeng Road, Wuxi 214000, China; 9862019007@jiangnan.edu.cn; 3 School of Food Science and Technology, Jiangnan University, 1800 Lihu Avenue, Wuxi 214122, China

**Keywords:** MOTS-c, radiation pneumonitis, mitochondrial function, Nrf2, epithelial cells

## Abstract

Radiation pneumonitis (RP) is a prevalent and fatal complication of thoracic radiotherapy due to the lack of effective treatment options. RP primarily arises from mitochondrial injury in lung epithelial cells. The mitochondrial-derived peptide MOTS-c has demonstrated protective effects against various diseases by mitigating mitochondrial injury. C57BL/6 mice were exposed to 20 Gy of lung irradiation (IR) and received daily intraperitoneal injections of MOTS-c for 2 weeks. MOTS-c significantly ameliorated lung tissue damage, inflammation, and oxidative stress caused by radiation. Meanwhile, MOTS-c reversed the apoptosis and mitochondrial damage of alveolar epithelial cells in RP mice. Furthermore, MOTS-c significantly inhibited oxidative stress and mitochondrial damage in MLE-12 cells and primary mouse lung epithelial cells. Mechanistically, MOTS-c increased the nuclear factor erythroid 2-related factor (Nrf2) level and promoted its nuclear translocation. Notably, Nrf2 deficiency abolished the protective function of MOTS-c in mice with RP. In conclusion, MOTS-c alleviates RP by protecting mitochondrial function through an Nrf2-dependent mechanism, indicating that MOTS-c may be a novel potential protective agent against RP.

## 1. Introduction

Radiation-induced lung injury (RILI) is a prevalent and inevitable complication of radiation therapy for thoracic tumors [1]. The pathogenesis of RILI remains intricate and not yet fully elucidated. It is widely accepted that RILI encompasses both early radiation pneumonitis (RP) and late radiation fibrosis [2]. Given the irreversible damage caused by late radiation fibrosis, prompt intervention is crucial in managing RP. However, the clinical application of amifostine, the only FDA-approved drug for radiological protection, has been limited due to its adverse side effects [3]. Therefore, it is imperative to develop novel therapeutic drugs for effectively treating RP.

Exposure of lung tissue to radiation often leads to the overproduction of reactive oxygen species (ROS), resulting in oxidative stress, inflammation, and cell apoptosis in alveolar epithelial cells. Mitochondria play a crucial role in this pathological process. Therefore, it is essential to mitigate radiation-induced mitochondrial damage and maintain mitochondrial homeostasis and function as strategies for ameliorating RP. Studies have revealed that MDPs play a key role in regulating various cellular processes such as apoptosis, inflammation, and oxidative stress, which are essential for maintaining cell viability [4]. In addition, MDPs also improve obesity and diabetes by regulating cellular metabolism. Short open-reading frames (sORF) in mitochondrial DNA has recently been found to encode micropeptides (peptides of no more than 100 amino acids) [5]. Three MDPs encoded by sORF have been identified, including humanin, small humanin-like peptides (SHLP), and MOTS-c. Among these MDPs, humanin and small humanin-like peptides (SHLP) are encoded by 16S ribosomal RNA, while MOTS-c is encoded by 12S ribosomal RNA [6]. MOTS-c is a recently discovered mitochondrial-encoded peptide consisting of 16 amino acids [7]. It is the first MDP demonstrated to enter the nucleus and enhance mitochondrial homeostasis through decreased oxygen consumption and ROS production while increasing mitochondrial membrane potential (Ψm) [8,9]. Recent studies have also indicated that MOTS-c protects against obesity, diabetes, osteoporosis, and aging through improved mitochondrial function [9,10,11,12]. With the increase of age, the expression level of MOTS-c in the human body gradually decreases, indicating that MOTS-c is closely related to aging [13]. Studies have shown that increased ROS production can promote cell aging [14]. Additionally, previous studies have demonstrated the anti-inflammatory effects of MOTS-c in various diseases, including Methicillin-resistant *staphylococcus aureus*-caused sepsis, formalin-induced inflammatory nociception, and LPS-induced acute lung injury [15]. However, it remains unknown whether MOTS-c can alleviate RP.

Nuclear factor erythroid-2 related factor 2 (Nrf2) is a transcription factor susceptible to oxidative stress and plays an important regulatory role in antioxidant lipid, heme, and iron metabolism. It binds to the antioxidant response element (ARE) in the nucleus and promotes the expression of various antioxidant genes such as NAD(P)H dehydrogenase quinone 1 (NQO1) and heme oxygenase-1 (HO-1) [16]. Recent studies have shown that Nrf2 activation can defend against mitochondrial toxins and ROS and affect mitochondrial function by regulating mitochondrial fatty acid oxidation, mitochondrial biogenesis, adenosine triphosphate (ATP) production, and mitochondrial dynamics [17,18,19]. In the mouse myocardium, Nrf2^−/−^ resulted in decreased expression of respiratory chain complex V and several mitochondrial DNA-encoded genes [20]. Nrf2^−/−^ mice have reduced complex I activity or reduced levels of proteins important for mitochondrial morphology in skeletal muscle [21]. Nrf2 activators have emerged as prospective therapeutic agents for the treatment of diseases caused by oxidative stress. Here, we propose that the MOTS-c peptide induces antioxidant and cytoprotective genes via Nrf2 upregulation and then attenuated RP. In the current study, X-ray irradiation decreased the expression of MOTS-c in mouse lung tissue, whereas exogenous MOTS-c supplementation significantly reduced RP. In addition, treatment with MOTS-c inhibited oxidative stress, inflammatory injury, and apoptosis in MLE-12 cells and primary mouse lung epithelial cells. Mechanism study revealed that MOTS-c promotes the expression and nuclear localization of Nrf2, enhances mitochondrial function, and inhibits epithelial cell damage; Nrf2 deficiency abolished the protective function of MOTS-c in mice with RP. These results imply that MOTS-c alleviates RP by protecting mitochondrial function through an Nrf2-dependent mechanism, indicating that MOTS-c may be a new agent against RP.

## 2. Materials and Methods

### 2.1. Animal Experimental Design

The animal experiments were approved by the Ethics Committee of Jiangnan University and conducted in accordance with established protocols. Pathogen-free male mice (6–8 weeks old) with a C57BL/6 background, including wild-type (WT) and Nrf2-knockout (Nrf2^−/−^) mice [22], were obtained from the Model Animal Research Center, MARC, Nanjing (project No. XM002783). The WT mice were randomly divided into three groups (*n* = 5): normal group (Normal), irradiation group (IR), and MOTS-c intervention group (IR+MOTS-c). Similarly, the Nrf2^−/−^ mice were also randomly divided into three groups (*n* = 5): normal group (Nrf2^−/−^), irradiation group (Nrf2^−/−^-IR), and MOTS-c intervention group (Nrf2^−/−^-IR+MOTS-c). Prior to irradiation, all mice were anesthetized using pentobarbital sodium at a dose of 40 mg/kg, and a whole-lung radiation dose of 20 Gy at a rate of 5 Gy/min was performed (Varian Vital Beam Linear Accelerator) [23]. Care was taken to avoid exposing the heart and spine as much as possible. Intraperitoneal injection of MOTS-c was administered 2 h before irradiation followed by daily injections for 13 days after radiation exposure [24]. The mice were sacrificed, and bronchoalveolar lavage fluid, blood samples, and lung tissue specimens were collected at 14 days post-irradiation.

### 2.2. Immunohistochemistry Staining

Sections of lung tissue, 4 µm thick, were deparaffinized, rehydrated, and subjected to antigen repair in 10 mM sodium citrate buffer heated to 95 °C for 30 min. Non-specific binding was blocked with normal goat serum for 20 min at 37 °C. The MOTS-c (#MOTSC-101AP, 1:100, FabGennix, Frisco, TX, USA) primary antibody was incubated overnight, followed by incubation with an HRP-labeled secondary antibody at room temperature for 1 h. Subsequently, 3, 3-diamino-benzidine (DAB) (Nanjing Jiancheng, Nanjing, China) was stained for 5 min and then counterstained with hematoxylin.

### 2.3. Histological Analysis

The left lungs of the mice were fixed with 4% paraformaldehyde by tracheal infusion and then immersed in the 4% paraformaldehyde solution for fixation. After the fixation, the left lung tissue specimen was embedded in paraffin and subjected to histopathologic examination. Tissue sections of 4 μm thickness were cut and stained with Haematoxylin and Eosin (HE) (Nanjing Jiancheng, Nanjing, China). We referred to another study for the lung injury score, which mainly has three criteria (inflammation, edema and hemorrhage, alveolar septal thickening). According to the severity of these criteria, it is graded from 0 to 4. The lung injury score is the sum of these three criteria. Detailed scoring is shown in the following Table 1 [25].

### 2.4. Collection and Analysis of Bronchoalveolar Lavage Fluid

Bronchoalveolar lavage fluid (BALF) was collected by intratracheal instillation with 0.6 mL of ice-cold 1 × PBS thrice and centrifuged at 500 g/min at 4 °C for 5 min. The supernatant was used to quantify BALF protein contents with a BCA protein assay kit (Yeasen, Shanghai, China), and the precipitates were counted and analyzed by flow cytometry (BD Accuri C6, Franklin Lakes, NJ, USA).

### 2.5. RNA-Sequence Data Acquirement

The transcriptomic data of GSE41789 were downloaded from the GEO database. In this dataset, differences in mRNA expression levels between three normal lung samples and three radiation pneumonitis samples were analyzed. The sangerbox was used to create a volcano map of differentially expressed genes.

### 2.6. Cell Cultures and Reagents

The mouse lung epithelial cells MLE-12 (ATCC, Manassas, VA, USA) were cultured in DMEM medium (Gibco, Grand Island, NY, USA) supplemented with 10% FBS (Yeasen, Shanghai, China) and 1% penicillin/streptomycin (Gibco, Grand Island, NY, USA). The cells were incubated in a cell culture chamber at 37 °C with 5% CO_2_. MLE-12 cells were seeded at a concentration of 1 × 10^5^/mL in 12-well plates or at a concentration of 2 × 10^5^/mL in 6-well plates. The final concentrations of the drugs used for cell treatment were as follows: MOTS-c (10 μM), ML385 (20 μM), MG132 (20 μM), and CHX (25 μM), respectively [26,27,28,29].

### 2.7. Biochemical Indexes Analysis

The biochemical indexes including myeloperoxidase (MPO), malondialdehyde (MDA), superoxide dismutase (SOD), glutathione (GSH), and lactate dehydrogenase (LDH) assays were detected by using commercial reagent kits (Nanjing Jiancheng, Nanjing, China) with an UV–VIS spectrophotometer (Thermo, Waltham, MA, USA) according to the manufacturer’s instructions.

### 2.8. Quantitative Real-Time PCR

The total RNA from cells or tissues was extracted by Trizol reagent (Vazyme, Nanjing, China), and 1 μg extracted RNA was reverse transcribed into cDNA using the RNA PCR Kit (Yeasen, Shanghai, China). Quantitative PCR was carried out using SYBR^®^ Green RT PCR Master Mix (Yeasen, Shanghai, China) and a Light Cycler 480-II Real-Time PCR system (Roche, Basel, Switzerland). Relative RNA expression levels were calculated by applying the 2^−ΔΔCt^ method, and the GAPDH gene was used as an endogenous control gene for normalizing the expression of target genes. The primer sequences are shown in Table S1.

### 2.9. Western Blot

The cells were lysed using RIPA lysis buffer supplemented with protease inhibitors and phosphatase inhibitors, followed by centrifugation at 12,000 rpm for 30 min. Protein concentration in the supernatant was determined using a BCA protein kit and subsequently denatured at 100 °C for 10 min. The protein was separated on a 12.5% SDS-PAGE gel and transferred onto a nitrocellulose filter. The filter was then blocked with 5% skimmed milk powder for 2 h before being incubated overnight at 4 °C with the primary antibody. After washing the membrane with TBST, the filter was incubated with the corresponding secondary antibody at room temperature for 2 h. The protein level was detected using a Bio-Rad imaging system equipped with a chemiluminescent substrate (Tanon, Shanghai, China). The primary antibodies used were as follows: Cyt-c (#ET1610-16, rabbit monoclonal, HUABIO, Woburn, MA, USA, 1:1000), COX IV (#ET1701-63, rabbit monoclonal, HUABIO, 1:1000), COXI (#0807-3, rabbit polyclonal, HUABIO, 1:1000), OPA1(#66583-1-Ig, mouse monoclonal, proteintech, Rosemont, IL, USA, 1:1000), Caspase9 (#ET1603-27, rabbit monoclonal, HUABIO, 1:1000), Bcl2 (#ER1802-97, rabbit polyclonal, HUABIO, 1:1000), Bax (#ET1603-34, rabbit monoclonal, HUABIO, 1:1000), Nrf2 (#12721, rabbit monoclonal, Cell Signaling Technology, Danvers, MA, USA, 1:1000), HO-1 (#ab223349, mouse monoclonal, Abcam, Cambridge, MA, USA, 1:1000), and GAPDH (#60004-1-Ig, mouse monoclonal, proteintech, 1:5000).

### 2.10. Immunofluorescence Staining

Cells were fixed with 4% paraformaldehyde at room temperature for 15 min. Then, they were permeabilized with 0.1% Triton X-100 for 20 min. Subsequently, the fixed cells were washed with PBS and blocked with 5% goat serum at room temperature for 30 min. The corresponding fluorescent secondary antibodies were incubated at room temperature for 2 h. After washing with PBS, the cells were sealed using DAPI reagent (Beyotime, Shanghai, China) and imaged using a Zeiss LSM 880 laser confocal fluorescence microscope (Carl Zeiss, Oberkochen, Germany). Paraffin sections of lung tissue underwent a deparaffinization and rehydration processes before being subjected to antigen retrieval in heated sodium citrate buffer (10 mM) maintained at a temperature of 95 °C for 30 min. The follow-up procedure was the same as for cellular immunofluorescence.

### 2.11. TUNEL Staining

A one-step TUNEL Apoptosis Assay Kit (Abbkine, Beijing, China) was applied to detect lung tissue apoptosis according to the manufacturer’s instructions. Briefly, tissue sections were rehydrated in reduced alcohol and then incubated with protease K at room temperature for 30 min. The TUNEL reaction mixture was then added to each section and incubated at 37 °C for 1 h. The tissue sections were subsequently washed with PBS, stained with DAPI, and photographed by fluorescence microscope.

### 2.12. Flow Cytometry

After 24 h of irradiation, the apoptosis rate of MLE-12 was detected using an Annexin V-FITC/PI Apoptosis Detection Kit. MLE-12 cells were inoculated into 6-well plates (2 × 10^5^/well) and treated with 10 μM MOTS-c for 24 h. Then cells were collected and reacted with 5 μL Annexin V-FITC and 5 μL propyl iodide (PI) at room temperature and in the dark for 20 min. Apoptosis was detected by flow cytometry (BD Biosciences, San Jose, CA, USA).

### 2.13. Detection of Cytosolic ROS and Mitochondrial ROS in MLE-12 Cells

To detect cytosolic ROS, MLE-12 cells were incubated with 10 μM 2′,7′-dichlorodihydrofluorescein diacetate (DCFH-DA) (Nanjing Jiancheng, Nanjing, China) for 1 h and then washed with PBS. The fluorescence intensity was measured by a fluorescence spectrophotometer (the wavelength was 485 nm and the emission wavelength was 530 nm). For the detection of mitochondrial ROS, MLE-12 cells were stained with 5 μM of MitoSOX Red Mitochondrial Superoxide Indicator (Yeasen, Shanghai, China) for 10 min. After staining, cells were washed with PBS, and then cells were counterstained with DAPI for 10 min. The stained cells were observed and photographed on a confocal microscope.

### 2.14. Mitochondrial Membrane Potential

Mitochondrial membrane potential (MMP) was measured using an Enhanced Mitochondrial Membrane Potential assay kit with JC-1 (Beyotime, Nanjing, China). Cells were seeded in 6-well plates and placed in an incubator overnight. Then, the cells were irradiated with 8 Gy and treated with 10 μM MOTS-c for 24 h. Subsequently, they were incubated with JC-1 staining working solution at 37 °C for 30 min and then washed three times with the JC-1 staining buffer. Photographs were then taken using a fluorescence microscope.

### 2.15. Adenosine Triphosphate Content

The intracellular adenosine triphosphate (ATP) content was quantified using an Enhanced ATP Assay Kit (Beyotime, Nanjing, China) following the manufacturer’s instructions. Briefly, cells were seeded in 6-well plates at a density of 2 × 10^5^ cells/well and cultured overnight. Subsequently, the cells were exposed to 8 Gy radiation and treated with MOTS-c at a concentration of 10 μM for 24 h. Lastly, cell lysis was performed using a lysis reagent followed by centrifugation at 12,000× *g* for 5 min at 4 °C. The supernatant was then collected to measure ATP levels.

### 2.16. Intracellular Calcium Concentration in MLE-12 Cells

Intracellular calcium levels of MLE-12 cells were analyzed using a Rhod-2 AM kit (Yeasen, Shanghai, China). The Rhod-2 AM fluorescent probe was diluted with HBSS without Ca^2+^ and Mg^2+^ at 1∶1000 to a final concentration of 5 μmol/L. Subsequently, the cells were incubated with a diluted Rhod-2 AM solution at 37 °C for 30 min and washed with HBSS three times. Finally, the cells were imaged using confocal microscopy.

### 2.17. Enzyme-Linked Immunosorbent Assay

The contents of MOTS-c and Cyt-c in mouse serum were measured with ELISA kits (Meimian, Yancheng, China).

### 2.18. Preparation of Primary AT II Cells

Primary alveolar epithelial type II (AT II) cells were isolated from mouse lungs as described previously. In brief, lung tissue was perfused with 0.25% trypsin (Sigma-Aldrich, St. Louis, MO, USA), aerated, and incubated at 37 °C. DNase (250 μg/mL; Sigma-Aldrich) passed the mixture through 300 μm and 40 μm filters (BD, Franklin Lakes, NJ, USA). After centrifugation, the cells were incubated in a humidified incubator at 37 °C for 2 h in a tissue culture flask. Non-adherent AT II cells in the suspension were removed and centrifuged. The cell pellet was resuspended in epithelial cell complete medium supplemented with 10% FBS and 1% penicillin-streptomycin solution. Surfactant protein C (SP-C; Abcam, Cambridge, MA, USA) [30].

### 2.19. Statistical Analysis

Measurement data were presented as means ± standard deviation (SD) of three independent experiments. The statistical significance between the two groups was assessed with the Student’s *t*-test. Multiple group comparisons were carried out by one-way analysis of variance (one-way ANOVA) followed by Tukey’s post-hoc analysis. *p*-value < 0.05 was considered to be statistically significant.

## 3. Results

### 3.1. MOTS-c Alleviated the Oxidant Damage, Inflammation, and Lung Tissue Injury in Irradiated Mice

**Figure 1:**
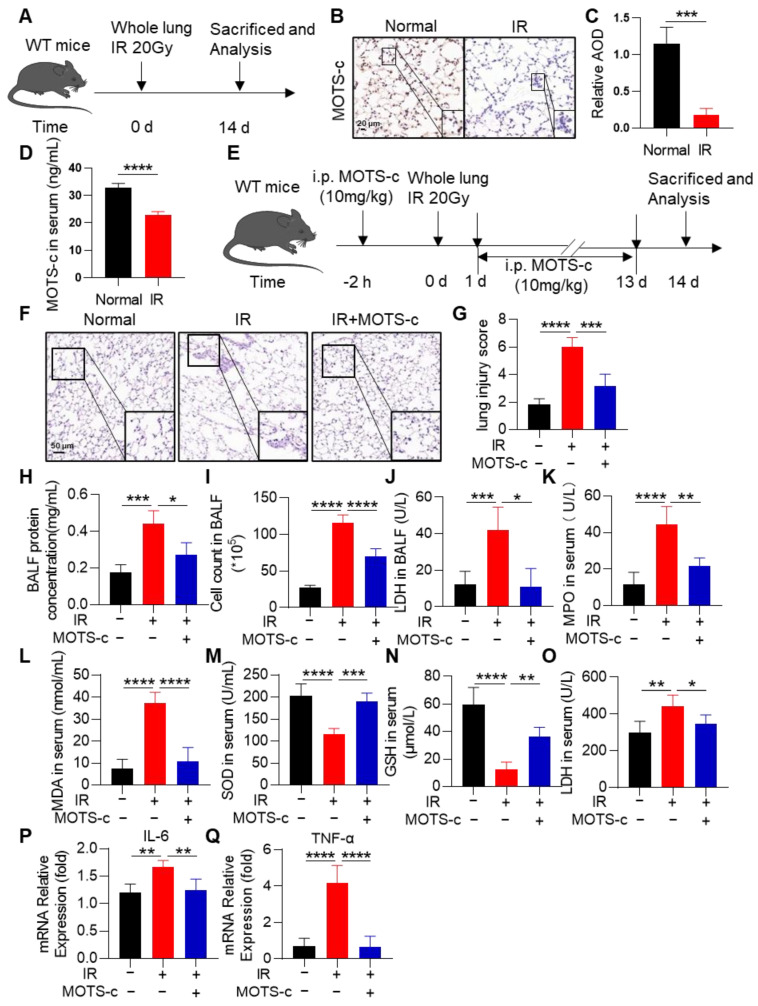
MOTS-c alleviated the oxidant damage, inflammation, and lung tissue injury in irradiated mice. (**A**) The schematic timeline of radiation-induced RP in WT mice. (**B**,**C**) Immunohistochemical staining was used to detect the expression of MOTS-c in lung tissue, scale bar = 20 μm. (**D**) ELISA was used to detect the expression of MOTS-c in serum. (**E**) Schematic timeline of MOTS-c intervention for radiation-induced RP in WT mice. Mice were anesthetized with pentobarbital sodium (40 mg/kg) and administered a dose of 20 Gy irradiation. After X-ray irradiation, mice in the MOTS-c intervention group received intraperitoneal injections of 10 mg/kg MOTS-c peptide dissolved in ddH_2_O daily for 14 days. (**F**) Lung tissue sections were stained with hematoxylin-eosin (HE), scale bar = 50 μm. (**G**) The lung injury score was calculated by histological analysis in different groups. (**H**,**I**) Total protein and cells in the BALF were detected. (**J**) LDH in the BALF was determined. (**K**–**O**) MPO, MDA, SOD, GSH, and LDH in mouse serum were determined. (**P**,**Q**) mRNA levels of *IL-6* and *TNF-α* were measured by quantitative real-time PCR. *n* = 5, * *p* < 0.05, ** *p* < 0.01, *** *p* < 0.001, **** *p* < 0.0001. IR: irradiation.

To investigate the impact of radiation on MOTS-c expression, mice were exposed to a dose of 20 Gy radiation (Figure 1A). Immunohistochemistry results demonstrated a significant decrease in MOTS-c protein content in the irradiated (IR) group (Figure 1B,C). Additionally, ELISA analysis revealed that radiation exposure reduced MOTS-c protein levels in mouse serum (Figure 1D). Furthermore, no significant effects on organ morphology or body weight were observed with MOTS-C treatment, indicating its low-toxicity advantage (Figure S1A,B). To further explore the therapeutic effect of MOTS-c in radiation pneumonitis (RP), mice were treated with radiation to induce RP with or without administration of MOTS-c (Figure 1E). Mice in the IR group exhibited severe morphological injuries such as edema, alveolar rupture, and infiltration of inflammatory cells into the parenchyma; however, these injuries were remarkably reduced with MOTS-c treatment (Figure 1F,G). Analysis of bronchoalveolar lavage fluid showed that MOTS-c significantly decreased cell count and protein concentration in BALF (Figure 1H,I), along with a reduction in LDH level upon administration of MOTS-c (Figure 1J). Moreover, we investigated oxidative status during radiation pneumonitis by examining MPO, MDA, SOD, GSH, and LDH levels in RP mice serum. Administration of MOTS-c remarkably reduced serum MPO, MDA, and LDH levels while significantly increasing SOD and GSH content (Figure 1K–O). Furthermore, mRNA expression levels of *IL-6* and *TNF-α* in lung tissue markedly decreased after MOTS-c treatment (Figure 1P,Q). These results showed that MOTS-c alleviated oxidant damage, inflammation, and lung tissue injury in mice with RP. In addition, we established an orthotopic lung cancer transplantation model and conducted X-ray irradiation to assess the impact of MOTS-c on tumor radiotherapy efficacy. The results demonstrated that MOTS-C did not enhance the proliferation of lung cancer cells following radiation therapy (Figure S1C–F). Collectively, these findings indicate that MOTS-c effectively mitigated RP in mice.

### 3.2. MOTS-c Prevented the Apoptosis of Alveolar Epithelial Cells in RP Mice

**Figure 2:**
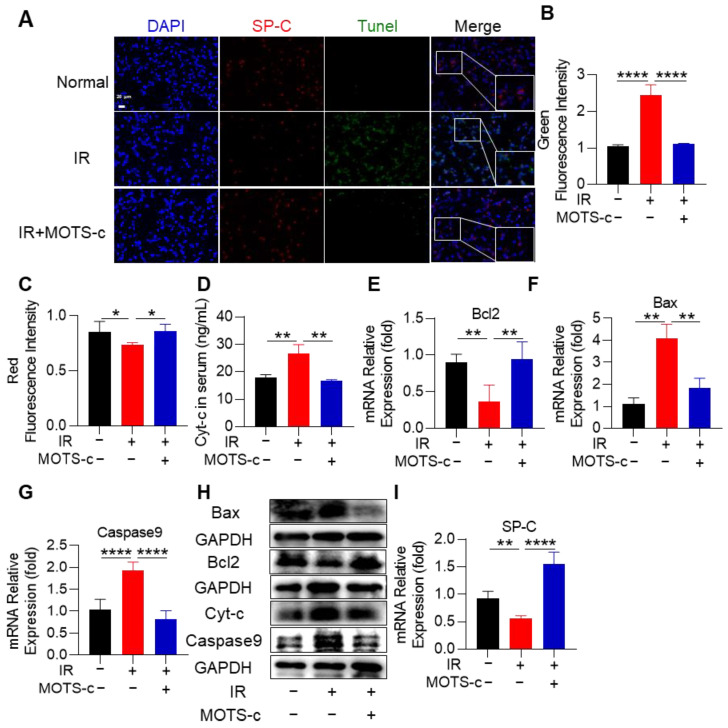
MOTS-c prevented the apoptosis of alveolar epithelial cells in RP mice. (**A**–**C**) Lung tissues were stained with the tunnel (green), SP-C (red), and DAPI (blue) for immunofluorescence analysis, scale bar = 20 μm. (**D**) ELISA was used to detect the expression of Cyt-c in serum. (**E**–**G**) mRNA levels of *Bcl2*, *Bax*, and *Caspase9* were measured by quantitative real-time PCR. (**H**) Protein levels of Cyt-c, Bcl2, Bax, and Caspase9 in lung tissues were assayed by western blotting. (**I**) mRNA level of SP-C was measured by quantitative real-time PCR. *n* = 5, * *p* < 0.05, ** *p* < 0.01, **** *p* < 0.0001. IR: irradiation.

Massive apoptosis of alveolar epithelial cells can result in severe radiation-induced pneumonia. As illustrated in Figure 2A–C, treatment with MOTS-c significantly attenuated the proportion of SP-C^+^ epithelial cells undergoing apoptosis in mice with RP. ELISA analysis demonstrated that MOTS-c treatment effectively reduced serum levels of Cytochrome c (Cyt-c) (Figure 2D). Subsequently, quantitative real-time PCR (qRT-PCR) was employed to assess the expression of key genes involved in the mitochondrial apoptosis pathway. As depicted in Figure 2E–G, MOTS-c treatment downregulated mRNA expression of pro-apoptotic genes *Bax* and *Caspase9* while upregulating mRNA expression of anti-apoptotic gene *Bcl2*. Furthermore, changes observed in protein content levels of Bcl2, Bax, and Caspase9 were consistent with alterations seen at the mRNA expression level (Figure 2H). Additionally, MOTS-c reversed radiation-induced reduction of SP-C mRNA levels (Figure 2I). Primary mouse lung epithelial cells were successfully isolated according to our previous study. Then, cells were treated with MOTS-c and exposed to irradiation. Results showed that MOTS-c significantly alleviated radiation-induced apoptosis and inflammation in epithelial cells (Figure S2A–E). Collectively, these findings indicate that MOTS-c inhibits apoptosis of alveolar epithelial cells in irradiated mice lungs.

### 3.3. MOTS-c Alleviates Radiation-Induced Oxidative Stress and Inflammation in MLE-12 Cells

**Figure 3:**
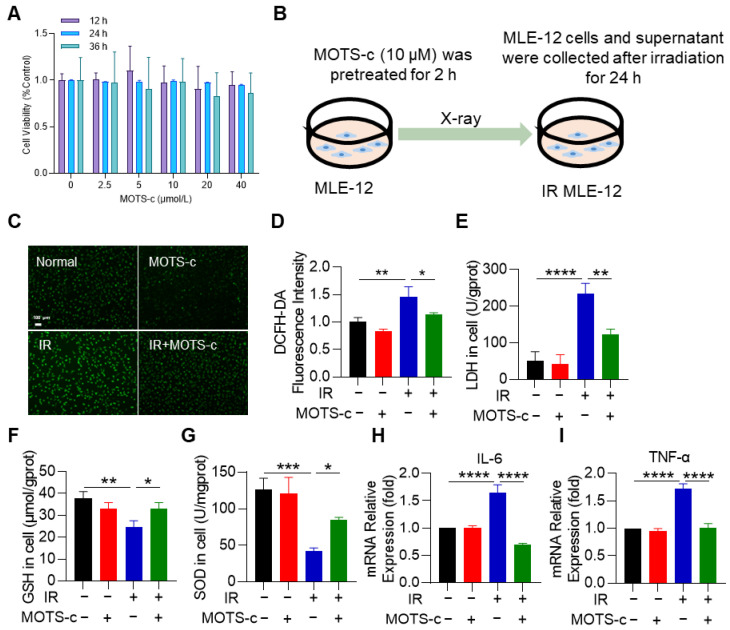
MOTS-c alleviates radiation-induced oxidative stress and inflammation in MLE-12 cells. (**A**) CCK8 assay was performed to detect the toxicity of MOTS-c to MLE-12. (**B**) Irradiation scheme for MLE-12 cells. (**C**,**D**) DCFH-DA fluorescent probes were used to detect the intracellular ROS, scale bar = 100 μm. (**E**–**G**) LDH, GSH, and SOD in MLE-12 were determined. (**H**,**I**) mRNA levels of *IL-6* and *TNF-α* were measured by qRT-PCR. *n* = 3, * *p* < 0.05, ** *p* < 0.01, *** *p* < 0.001, **** *p* < 0.0001. IR: irradiation.

The cytotoxicity of MOTS-c on MLE-12 cells was assessed using the CCK-8 assay. As illustrated in Figure 3A, MOTS-c did not exhibit significant toxicity towards MLE-12 cells. Following irradiation with 8 Gy X-rays and pretreatment with MOTS-c for 2 h, MLE-12 cells were cultured for an additional 24 h in subsequent experiments (Figure 3B). Pretreatment with MOTS-c at a concentration of 10 μM significantly attenuated ROS levels in MLE-12 cells (Figure 3C,D), while also reversing the radiation-induced elevation of LDH in the cell culture supernatant (Figure 3E). To investigate whether MOTS-c treatment conferred protection against radiation-induced oxidative stress, SOD and GSH levels were measured in MLE-12 cells. The results demonstrated that treatment with MOTS-c increased SOD and GSH levels (Figure 3F,G). Moreover, mRNA expression levels of *IL-6* and *TNF-α* were significantly reduced in MLE-12 cells treated with MOTS-c (Figure 3H,I), indicating its potential to mitigate radiation-induced oxidative stress and inflammatory response.

### 3.4. MOTS-c Reduced the Radiation-Induced Apoptosis of MLE-12 Cells

**Figure 4:**
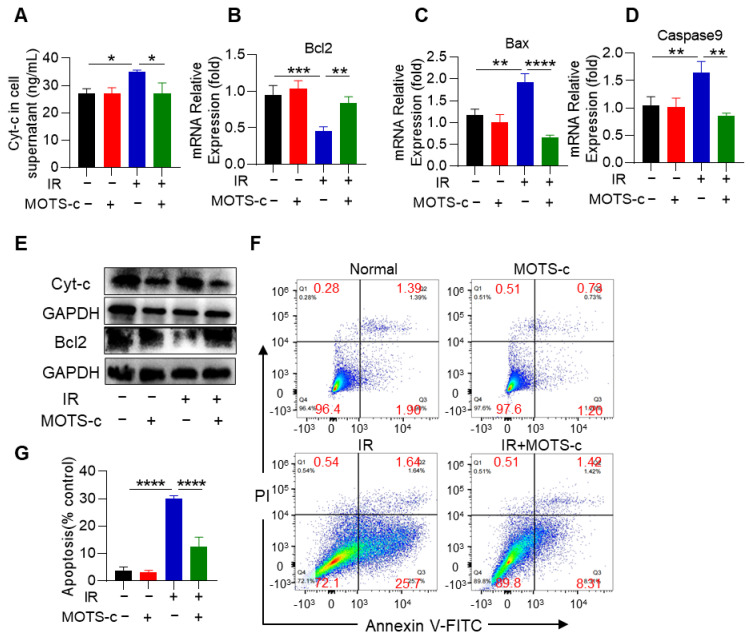
MOTS-c reduced the radiation-induced apoptosis of MLE-12 cells. (**A**) ELISA was used to detect the expression of Cyt-c in the cell supernatant. (**B**–**D**) mRNA levels of *Bcl2*, *Bax*, and *Caspase9* were measured by quantitative real-time PCR in MLE-12. (**E**) Protein levels of Cyt-c and Bcl2 in lung tissues were assayed by western blotting. (**F**,**G**) The apoptosis of MLE-12 was measured by flow cytometry. *n* = 3, * *p* < 0.05, ** *p* < 0.01, *** *p* < 0.001, **** *p* < 0.0001. IR: irradiation.

ELISA analysis demonstrated that MOTS-c significantly decreased Cyt-c levels in cell supernatant (Figure 4A). MOTS-c also significantly downregulated the mRNA expression of the pro-apoptotic genes *Bax* and *Caspase9*, while upregulating the anti-apoptotic gene *Bcl2* (Figure 4B–D). Moreover, MOTS-c treatment decreased the protein content of pro-apoptotic genes Cyt-c, while increasing the anti-apoptotic gene Bcl2 (Figure 4E). Flow cytometry also showed that MOTS-c significantly inhibited radiation-induced apoptosis in MLE-12 cells (Figure 4F,G).

### 3.5. MOTS-c Relieved Radiation-Induced Mitochondrial Damage in MLE-12 Cells

**Figure 5:**
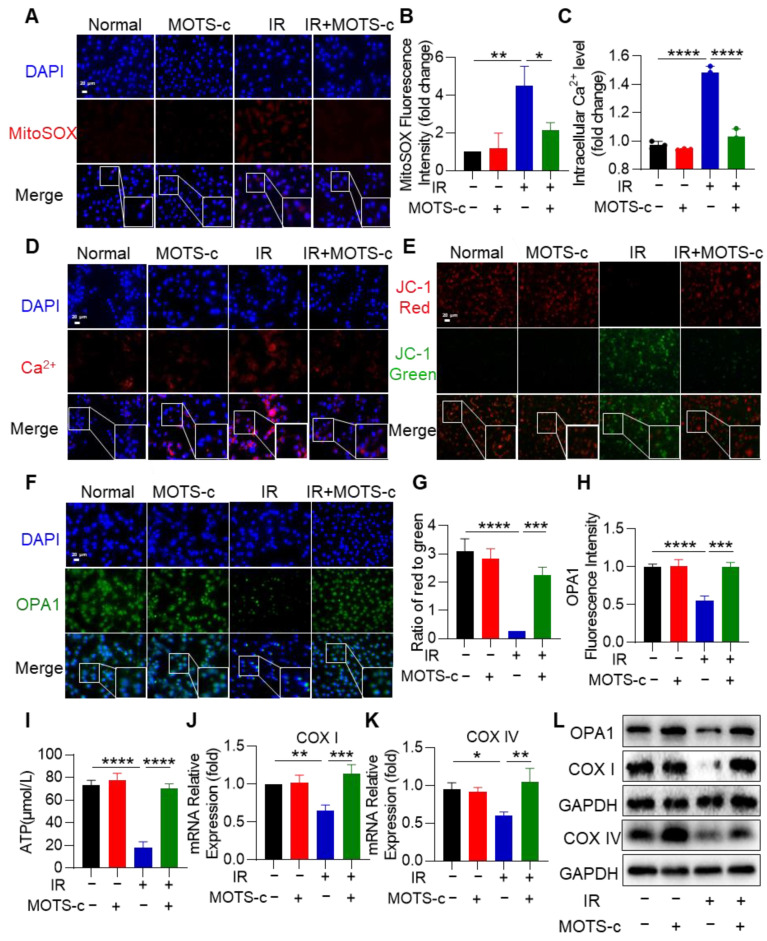
MOTS-c relieved radiation-induced mitochondrial damage in MLE-12 cells. (**A**,**B**) MitoSOX Red fluorescent probe (5 μM) was used to detect mitochondrial reactive oxygen species in MLE-12, scale bar = 20 μm. (**C**,**D**) Rhod-2, AM Red fluorescent probe (4 μM) was used to detect calcium content in MLE-12, scale bar = 20 μm. (**E**,**G**) Mitochondrial membrane potential of MLE-12 was detected by JC-1 staining kit, scale bar = 20 μm. (**F**,**H**) MLE-12 were stained with the OPA1 (green) and DAPI (blue) for immunofluorescence analysis, scale bar = 20 μm. (**I**) The content of ATP in MLE-12 was detected by the ATP detection kit. (**J**,**K**) mRNA levels of *COX I* and *COX IV* were measured by quantitative PCR. (**L**) Protein levels of COX I, COX IV, and OPA1 in MLE-12 were assayed by western blotting. *n* = 3, * *p* < 0.05, ** *p* < 0.01, *** *p* < 0.001, **** *p* < 0.0001. IR: irradiation.

To investigate the role of MOTS-c in mitigating radiation-induced mitochondrial damage, we conducted experiments to assess mitochondrial function. Analysis using fluorescence microscopy and confocal laser scanning microscopy revealed significantly weaker red fluorescent signals for MitoSOX and Rhod-2 AM in the IR+MOTS-c group (Figure 5A–D). Furthermore, MOTS-c prevented the decrease in the ratio of JC-1 aggregate (red) to JC-1 monomer (green) (Figure 5E,G). As shown in Figure 5F,H, MOTS-c increased OPA1 protein content. These results suggest that MOTS-c protects MLE-12 cells from radiation-induced mitochondrial damage. Additionally, radiation substantially decreased ATP production in MLE-12 cells, which was restored by supplementation with MOTS-c (Figure 5I). The mRNA expression levels of *COX I* and *COX IV* were remarkably increased after supplementation with MOTS-c in MLE-12 cells (Figure 5J,K). Moreover, supplementation with MOTS-c also led to an increase in protein contents of COX I, COX IV, and OPA1 (Figure 5L). Overall, these findings indicate that MOTS-c eliminated excessive ROS and preserved mitochondrial function in radiation-induced MLE-12 cells.

### 3.6. MOTS-c Mitigated Mitochondrial Injury in RP Mice

**Figure 6:**
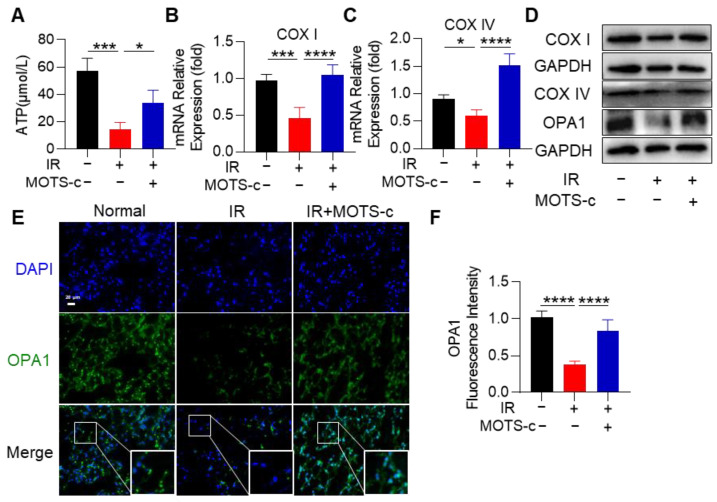
MOTS-c mitigated mitochondrial injury in RP mice. (**A**) ATP detection kit was used to detect the content of ATP in lung tissue. (**B**,**C**) mRNA levels of *COX I* and *COX IV* were measured by quantitative real-time PCR in lung tissue. (**D**) The expression of OPA1, COX I, and COX IV were determined using the western blot. (**E**,**F**) Lung tissues were stained with the OPA1 (green) and DAPI (blue) for immunofluorescence analysis, scale bar = 20 μm. *n* = 5, * *p* < 0.05, *** *p* < 0.001, **** *p* < 0.0001. IR: irradiation.

To further confirm the beneficial role of MOTS-c in RP mice, we detected mitochondrial function in lung tissue. MOTS-c increased ATP production, which was decreased by radiation exposure (Figure 6A). The mRNA expression levels of *COX I* and *COX IV* in lung tissue were remarkably increased after MOTS-c treatment (Figure 6B,C). In addition, the protein content of COX I, COX IV, and OPA1 was also increased after treatment with MOTS-c (Figure 6D). Subsequently, immunofluorescence staining of lung tissues revealed that MOTS-c reversed the radiation-induced reduction in OPA1 protein content (Figure 6E,F). Taken together, these results suggest that MOTS-c protected mitochondrial function in RP mice.

### 3.7. MOTS-c Increased Nrf2 Content and Induced Its Nucleus Translocation in RP Mice

**Figure 7:**
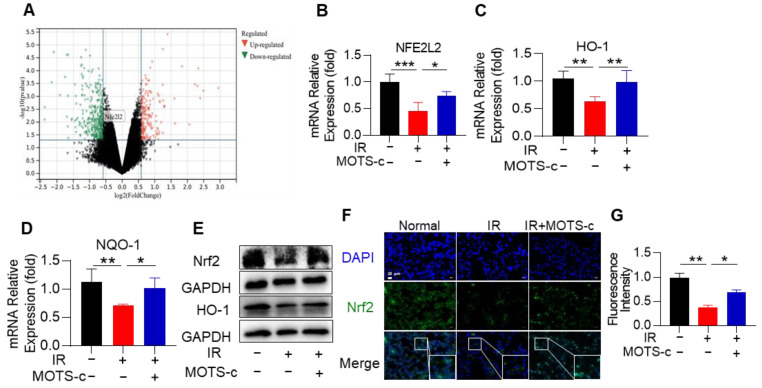
MOTS-c increased Nrf2 content and induced its nucleus translocation in RP mice. (**A**) The volcano map showed that the expression of Nrf2 in mice was decreased after irradiation. (**B**–**D**) The mRNA levels of *NFE2L2*, *HO-1*, and *NQO1* were measured by quantitative real-time PCR in lung tissue. (**E**) The expression of Nrf2, and HO-1 were determined using the western blot. (**F**,**G**) Lung tissues were stained with the Nrf2 (green) and DAPI (blue) for immunofluorescence analysis, scale bar = 20 μm. *n* = 5, * *p* < 0.05, ** *p* < 0.01, *** *p* < 0.001. IR: irradiation.

The public volcano map databases showed that the expression of Nrf2 in mice was decreased after irradiation (Figure 7A). Considering Nrf2 plays an important role in RP, we questioned whether MOTS-c exerts a protective effect by targeting Nrf2. MOTS-c significantly increased the mRNA expression levels of *Nrf2*, *HO-1*, and *NQO-1* (NADPH: qunone qxidoreductase 1) in mice with RP (Figure 7B–D). As displayed in Figure 7E, MOTS-c remarkably increased the protein content of Nrf2 and its downstream target gene HO-1 in lung tissue. Furthermore, immunofluorescence results demonstrated that MOTS-c could significantly promote the nuclear translocation of Nrf2 (Figure 7F,G).

### 3.8. MOTS-c Increased Nrf2 Content and Induced Its Nucleus Translocation in MLE-12 Cells

**Figure 8:**
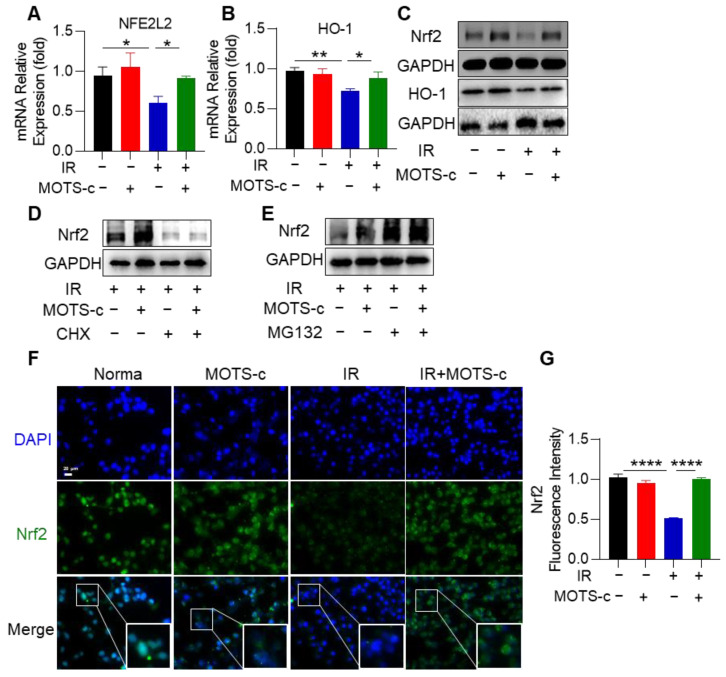
MOTS-c promoted Nrf2 synthesis and translocation to the nucleus. (**A**,**B**) mRNA levels of *NFE2L2* and *HO-1* were measured by quantitative real-time PCR in MLE-12. (**C**) Protein levels of Nrf2 and HO-1 in MLE-12 were assayed by western blotting. (**D**,**E**) MLE-12 was pretreated with 10 μM MOTS-c for 2 h following radiation 24 h, then treated with CHX (25 μM) or MG132 (20 μM). The expression of Nrf2 was determined using the western blot. (**F**,**G**) MLE-12 cells were stained with the Nrf2 (green) and DAPI (blue) for immunofluorescence analysis, scale bar = 20 μm. *n* = 3, * *p* < 0.05, ** *p* < 0.01, **** *p* < 0.0001. IR: irradiation.

To investigate the molecular mechanisms underlying the protective effects of MOTS-c against radiation injury, we conducted an analysis of a public database and observed downregulation of the transcription factor Nrf2 in RP mice (Figure S2A). Subsequent experiments focused on exploring the relationship between MOTS-c and Nrf2 in relation to RP. As shown in Figure 8A,B, MOTS-c significantly increased mRNA expression levels of *NFE2L2* (Nrf2) as well as its downstream target gene *HO-1* in MLE-12 cells. Western blot results were consistent with those obtained from qRT-PCR analysis (Figure 8C). Furthermore, pretreatment with both MOTS-c and radiation followed by CHX treatment demonstrated that CHX nullified the upregulatory effect of MOTS-c on Nrf2 (Figure 8D). Additionally, MG132 was found to enhance the protein content of Nrf2 upon treatment with MOTS-c, indicating that MOTS-c could directly promote synthesis of Nrf2 independent of protein degradation (Figure 8E). Immunofluorescence staining revealed that MOTS-c significantly facilitated translocation of Nrf2 into the nucleus within MLE-12 cells (Figure 8F,G). These findings collectively suggest that MOTS-c increased Nrf2 content and promoted its nuclear translocation in MLE-12 cells.

### 3.9. Inhibition of Nrf2 Abolished the Protective Function of MOTS-c in MLE-12 Cells

**Figure 9:**
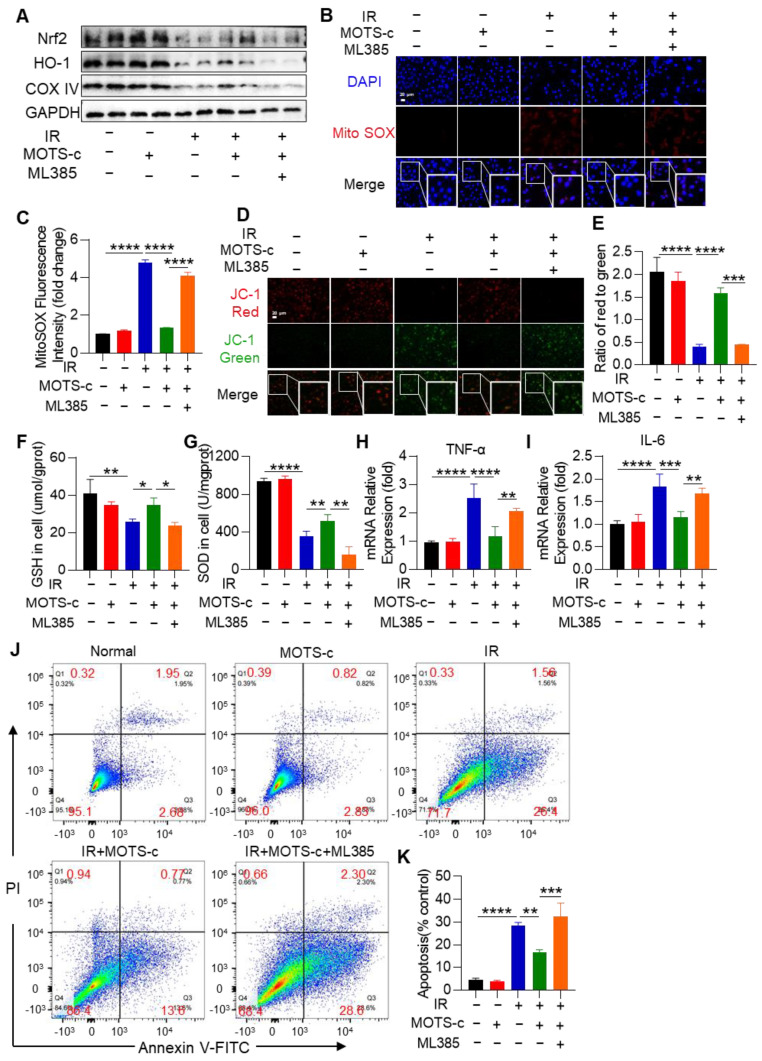
Inhibition of Nrf2 abolished the protective function of MOTS-c in MLE-12 cells. (**A**) To further evaluate whether MOTS-c plays a protective role through Nrf2, cells were pretreated with Nrf2 inhibitor ML385. The expression of Nrf2, HO-1, and COX IV was determined using the western blot. (**B**,**C**) MitoSOX Red fluorescent probe (5 μM) was used to detect mitochondrial reactive oxygen species in MLE-12, scale bar = 20 μm. (**D**,**E**) Mitochondrial membrane potential of MLE-12 was detected by JC-1 staining kit, scale bar = 20 μm. (**F**,**G**) GSH and SOD in MLE-12 were determined. (**H**,**I**) The mRNA levels of *IL-6* and *TNF-α* in MLE-12 were measured by quantitative real-time PCR. (**J**,**K**) The apoptosis of MLE-12 was measured by flow cytometry. *n* = 3, * *p* < 0.05, ** *p* < 0.01, *** *p* < 0.001, **** *p* < 0.0001. IR: irradiation.

To further investigate the protective role of MOTS-c on IR-induced epithelial cell damage through the Nrf2 pathway, cells were pretreated with the Nrf2 inhibitor ML385 (Figure S3A). The results demonstrated that pretreatment with ML385 diminished the increased effect of MOTS-c on COX4 and HO-1 protein content (Figure 9A). Additionally, MOTS-c did not reduce MitoSOX fluorescence intensity or calcium levels after ML385 treatment, as shown in Figure 9B,C and Figure S3B,C. Furthermore, in the IR+MOTS-c+ML385 group, MOTS-c lost its protective effect on OPA1 protein expression and mitochondrial membrane potential (Figure S3D,E and Figure 9D,E). Moreover, MOTS-c treatment did not mitigate radiation-induced reductions in GSH and SOD levels after ML385 treatment (Figure 9F,G). The mRNA expression levels of *IL-6* and *TNF-α* were also not reduced by MOTS-c after ML385 treatment (Figure 9H,I). Flow cytometry results indicated that the anti-apoptotic effect of MOTS-c on MLE-12 cells was abolished by ML385 (Figure 9J,K). These findings suggest that inhibition of Nrf2 eliminates the protective role of MOTS-c against radiation damage in MLE-12 cells.

### 3.10. Nrf2 Deficiency Abolished the Protective Function of MOTS-c in RP Mice

**Figure 10:**
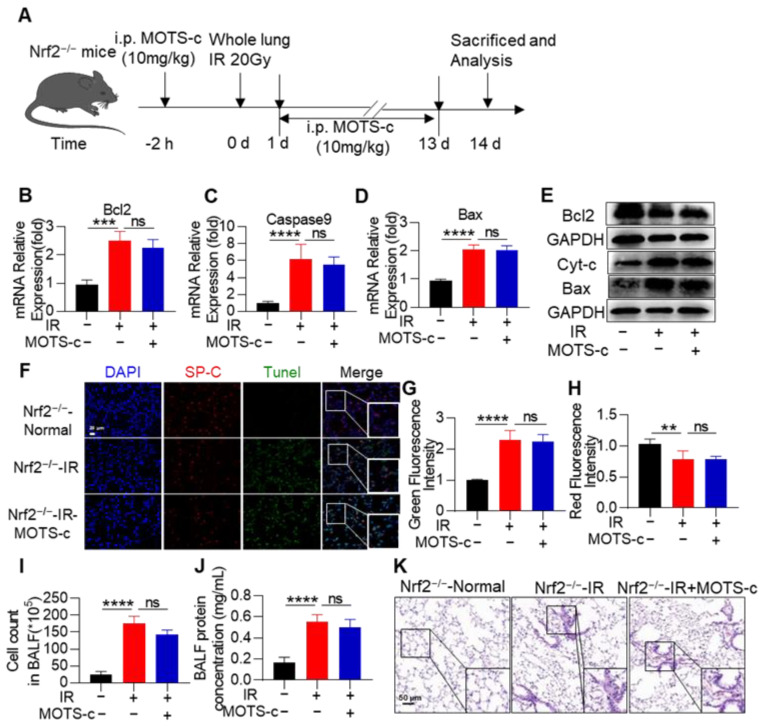
Nrf2 deficiency abolished the protective function of MOTS-c on RP mice. (**A**) Schematic timeline of MOTS-c intervention for radiation-induced PR in Nrf2^−/−^ mice. (**B**–**D**) The mRNA levels of *Bcl2*, *Bax*, and *Caspase9* in lung tissues were measured by quantitative real-time PCR. (**E**) Protein levels of Cyt-c, Bcl2, and Bax in lung tissues were assayed by western blotting. (**F**–**H**) Lung tissues were stained with the tunnel (Green), SP-C (Red), and DAPI (blue) for immunofluorescence analysis, scale bar = 20 μm. (**I**,**J**) Total protein and cells in the BALF were detected. (**K**) Lung tissue sections were stained with hematoxylin-eosin (HE), scale bar = 100 μm. *n* = 5, ns means no significant difference, ** *p* < 0.01, *** *p* < 0.001, **** *p* < 0.0001. IR: irradiation.

Confirming the role of Nrf2 in the protective effect of MOTS-c against RP, we conducted experiments using Nrf2^−/−^ mice (Figure S4 and Figure 10A). Administering MOTS-c did not lead to a decrease in levels of MDA and LDH or an increase in SOD content in the serum of irradiated Nrf2^−/−^ mice (Figure S5A–C). Additionally, deletion of Nrf2 reduced the inhibitory effect of MOTS-c on the expression levels of pro-inflammatory cytokines IL-6 and TNF-α (Figure S5D,E) and apoptosis-related genes (Figure 10B–E). In Nrf2^−/−-^ mice with radiation pneumonitis, MOTS-c was ineffective in reversing the apoptosis of SP-C^+^ epithelial cells (Figure 10F,G), reducing cell infiltration, or protein leakage into BALF (Figure 10I,J). Histopathological examinations revealed severe lung tissue damage in Nrf2 knockout mice exposed to radiation, and MOTS-c did not alleviate these structural abnormalities (Figure 10K). Overall, these results indicate that the protective effects of MOTS-c against radiation pneumonitis are mediated through Nrf2.

## 4. Discussion

Radiation therapy often results in damage to normal lung tissue, presenting a significant challenge to its efficacy. It is crucial to find ways to mitigate or prevent adverse effects on the lungs caused by radiation therapy [31,32]. Our study shows that radiation induces the overproduction of ROS, leading to mitochondrial damage in a mouse model of RP. However, administration of MOTS-c, a mitochondria-derived peptide, can alleviate this detrimental effect. Additionally, MOTS-c protects against pulmonary inflammation, oxidative stress, and epithelial cell apoptosis in RP by preserving mitochondrial function. Through mechanistic investigations, we have determined that MOTS-c exerts its protective effects by activating the Nrf2 signaling pathway. Overall, our results suggest that MOTS-c mitigates RP by improving mitochondrial function through an Nrf2-dependent mechanism.

Previous studies have demonstrated that both single high-dose and multiple low-dose radiation exposures can effectively establish mouse models of radiation pneumonia [33,34,35,36]. These methods accurately reflect the pathophysiological changes observed in patients undergoing radiotherapy for lung injury. Considering the advantages of convenience and efficiency associated with single high-dose irradiation, we exposed mice to a radiation dose of 20 Gy in this study. Consequently, the mice exhibited characteristic indicators of radiation pneumonia such as edema, alveolar rupture, and infiltration of inflammatory cells into the parenchyma. These findings provide evidence supporting the successful establishment of a mouse model for studying radiation-induced pneumonia.

The key finding of this study is that MOTS-c exerts a protective effect on mitochondria, thereby attenuating RP in mice. Given the vulnerability of mitochondria to radiation, damaged mitochondria and their respiratory chain complexes are implicated in the overall process of RP. Radiation induces DNA damage or mutations in mitochondria and reduces mitochondrial RNA transcription [37]. MOTS-c maintains mitochondrial functional homeostasis and promotes mitochondrial biosynthesis in various diseases such as aging, cardiovascular disease, and insulin resistance [37]. In both in-vivo and in-vitro studies conducted here, radiation exposure led to increased ROS production, inhibited ATP production, and decreased expression of OPA1, COX I, and COX IV proteins—indicative of severe mitochondrial damage. Treatment with MOTS-c significantly reduced serum levels of MPO, MDA, and LDH, while increasing SOD activity and GSH content. Furthermore, administration of MOTS-c markedly suppressed total ROS and mtROS generation, enhanced mitochondrial membrane potential, and upregulated expression of OPA1, COX I,and COX IV proteins. These findings underscore the beneficial role of MOTS-c against RP in mice.

The role of alveolar epithelial cells in maintaining lung homeostasis is crucial, but they are often damaged by radiotherapy and undergo apoptosis. Previous studies have shown that normal lung tissue can undergo apoptosis immediately after and for several weeks following radiation exposure [38]. Apoptosis pathways in cells primarily involve endogenous mitochondrial, exogenous death receptor, and endoplasmic reticulum pathways [39]. Mitochondria play a central role in energy production and apoptosis in most eukaryotic cells [40]. Mitochondrial dysfunction, characterized by excessive ROS production, insufficient ATP production, and increased mitochondrial fragmentation, can lead to apoptosis. MitoQ, a mitochondrial-targeted antioxidant, has been shown to protect against acute lung injury in rodents by preserving mitochondrial homeostasis and inhibiting endothelial cell apoptosis [41]. Maintaining mitochondrial homeostasis has been proposed as a potential treatment for RP. In this study, we found that MOTS-c significantly reduced apoptosis of SP-C^+^ epithelial cells in RP mice. The expression of pro-apoptotic factors such as BAX, Caspase9, and Cyt-c protein was increased in lung tissue following radiation exposure, while the expression of the anti-apoptotic gene Bcl2 was decreased. However, MOTS-c reversed these radiation-induced changes in apoptosis-related genes. Additionally, MOTS-c protected lung epithelial cells from apoptosis and improved mitochondrial quality. ELISA results showed that MOTS-c could reduce the radiation-induced increase in serum Cyt-c levels. By regulating the expression of Cyt-c, Bcl2, BAX, and Caspase9, MOTS-c inhibited the mitochondrial death pathway and significantly reduced epithelial cell apoptosis. Consistent with our findings, MOTS-c has been reported to inhibit ERK1/2 activation and EGR1 expression by downregulating the CCN1 gene, thereby protecting mitochondrial function and reducing myocardial cell apoptosis [41]. These results suggest that MOTS-c is a promising anti-apoptotic agent against RP.

RP is characterized by severe inflammation in normal lung tissue, which occurs as a result of cell injury to the alveolar and vascular epithelium after exposure to ionizing radiation. This leads to recruitment of inflammatory cells that secrete pro-inflammatory cytokines and adhesion molecules. Additionally, persistent inflammation stimulates lung fibroblast proliferation and extracellular matrix secretion, resulting in irreversible stiffening of the alveolar walls and respiratory dysfunction [42,43,44]. However, frequent use of anti-inflammatory drugs like amifostine and glucocorticoids can have serious side effects such as digestive and cardiovascular complications. MOTS-c has been widely reported for its anti-inflammatory effect in various disease models, including chronic kidney disease, acute and neuropathic pain, osteolysis, and acute lung injury [45,46,47,48]. MOTS-c treatment significantly decreased the transcript levels of inflammatory factors (IL-1β, IL-4, IL-6, and TNF-α) as well as circulating myocardial injury markers (CK-MB and TnT) [49]. Furthermore, MOTS-c was shown to enhance macrophage phagocytosis and increase expression of anti-inflammatory regulators [15]. Our study demonstrated the following after MOTS-c treatment: (1) mRNA expression levels of IL-6andTNF-α in lung tissue were remarkably decreased; (2) the levels of IL-6 and TNF-α mRNA were significantly decreased in MLE12 cells after MOTS-c pretreatment; (3) MOTS-c effectively reduced the cell number and protein concentration in the BALF. Additionally, MOTS-c had no effect on normal mice nor negatively affected the efficacy of tumor radiotherapy. These experimental results suggest that MOTS-c is a potential anti-inflammatory agent for RP therapy.

MOTS-c interacts with various transcription factors, including FOXF1, Runx2 [50], Nrf2 [51], STAT3 [15], and NF-κB [52]. However, it remains unclear which pathways are responsible for the protective effect of MOTS-c against RP. One known pathway is the Nrf2/ARE pathway, which is associated with anti-oxidative, anti-apoptotic, and anti-inflammatory effects. Patients with chronic kidney disease exhibit reduced levels of mitochondria, MOTS-c, and Nrf2 in their renal tissue, leading to chronic inflammation and oxidative stress [47]. In H9c2 cells induced by H_2_O_2_, MOTS-c exerts a protective effect on inflammatory response and oxidative stress by inhibiting NF-κB activation while activating the Nrf2/ARE pathway [53]. These findings suggest that MOTS-c/Nrf2 may play a crucial role in combating inflammation and oxidative stress. To investigate the role of Nrf2 in the protective effect of MOTS-c against RP, we conducted experiments using CHX (cycloheximide), MG132 (proteasome inhibitor), ML385 (Nrf2 inhibitor), as well as Nrf2-knockout RP rodents. Both in-vitro and in-vivo models demonstrated that MOTS-c significantly increased mRNA expression levels of NFE2L2 (Nrf2 gene) and its downstream target genes HO-1 (heme oxygenase 1) and NQO-1 (NAD(P)H quinone dehydrogenase 1). Furthermore, immunofluorescence results confirmed that MOTS-c effectively promoted nuclear translocation of Nrf2. Notably, when tested on mice lacking functional Nrf2 genes or MLE-12 cells with inhibited expression of Nrf2 due to ML385 treatment, MOTS-c failed to exert its protective effect against apoptosis. Therefore, we proposed that MOTS-c protects against RP involves activation of the Nrf2/ARE signaling pathway via direct promotion synthesis rather than degradation.

We acknowledge that the present study has three limitations. Additional work will be required to obtain samples from clinical patients for analysis in the future. Moreover, Nrf2 can promote the transcription of more than 400 genes, and the specific pathway through which to achieve mitochondrial protection remains to be confirmed. In addition, Nrf2 epithelial specific knockout mice need to be further established for validation.

## 5. Conclusions

**Figure 11:**
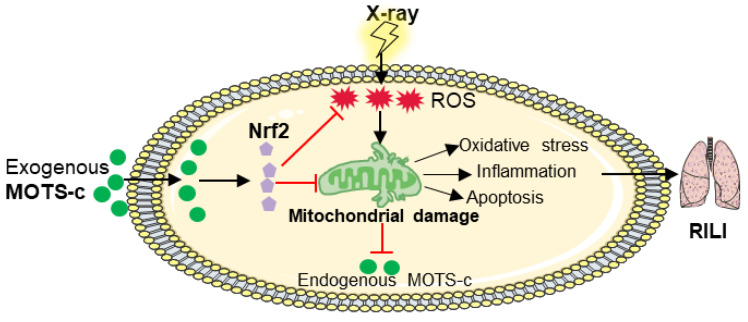
Potential mechanism of MOTS-c exerting a radioprotective effect on RILI.

In conclusion, we firstly found that MOTS-c can reduce mitochondrial ROS, maintain mitochondrial homeostasis, and inhibit epithelial apoptosis via Nrf2/ARE signaling (Figure 11). As an endogenous peptide, MOTS-c may be safer and more effective than exogenous medications. These findings suggest a novel function for MOTS-c as a prospective drug treatment for RP.

## Figures and Tables

**Table 1:** Criteria of lung injury score.

Criteria	Score = 0	Score = 1	Score = 2	Score = 3	Score = 4
Inflammation	Absent	Mild	Moderate	Severe	Very severe
Edema and hemorrhage	None	<10%	10–30%	30–50%	>50%
Alveolar septal thickening	<15 μm	15–30 μm	30–45 μm	45–60 μm	>60 μm

## Data Availability

The original data are available from the corresponding author upon reasonable request.

## Supplementary Materials

The following supporting information can be downloaded at: https://www.mdpi.com/article/10.3390/antiox13050613/s1, Figure S1: Effect of MOTS-c on different organs of normal mice and tumor radiotherapy of orthotopic transplantation of lung cancer mice; Figure S2: MOTS-c prevented the apoptosis of alveolar epithelial cells in RP mice; Figure S3: MOTS-c relieved radiation-induced mitochondrial damage in MLE-12 cells; Figure S4: Gene identification results in WT and Nrf2^−/−^ mice; Figure S5: Nrf2 deficiency abolished the protective function of MOTS-c on RP mice; Table S1: Primer sequences.
