# Supplementary material for: The Mitochondrial-Derived Peptide MOTS-c Alleviates Radiation Pneumonitis via an Nrf2-Dependent Mechanism
Source: Antioxidants (Basel). 2024 May 17;13(5):613. doi: 10.3390/antiox13050613 (PMC11117534; doi:10.3390/antiox13050613)
Supplement: Supplementary file 1 [file antioxidants-13-00613-s001.zip › Supplementary material and figures-20240509.pdf]

Figure S1

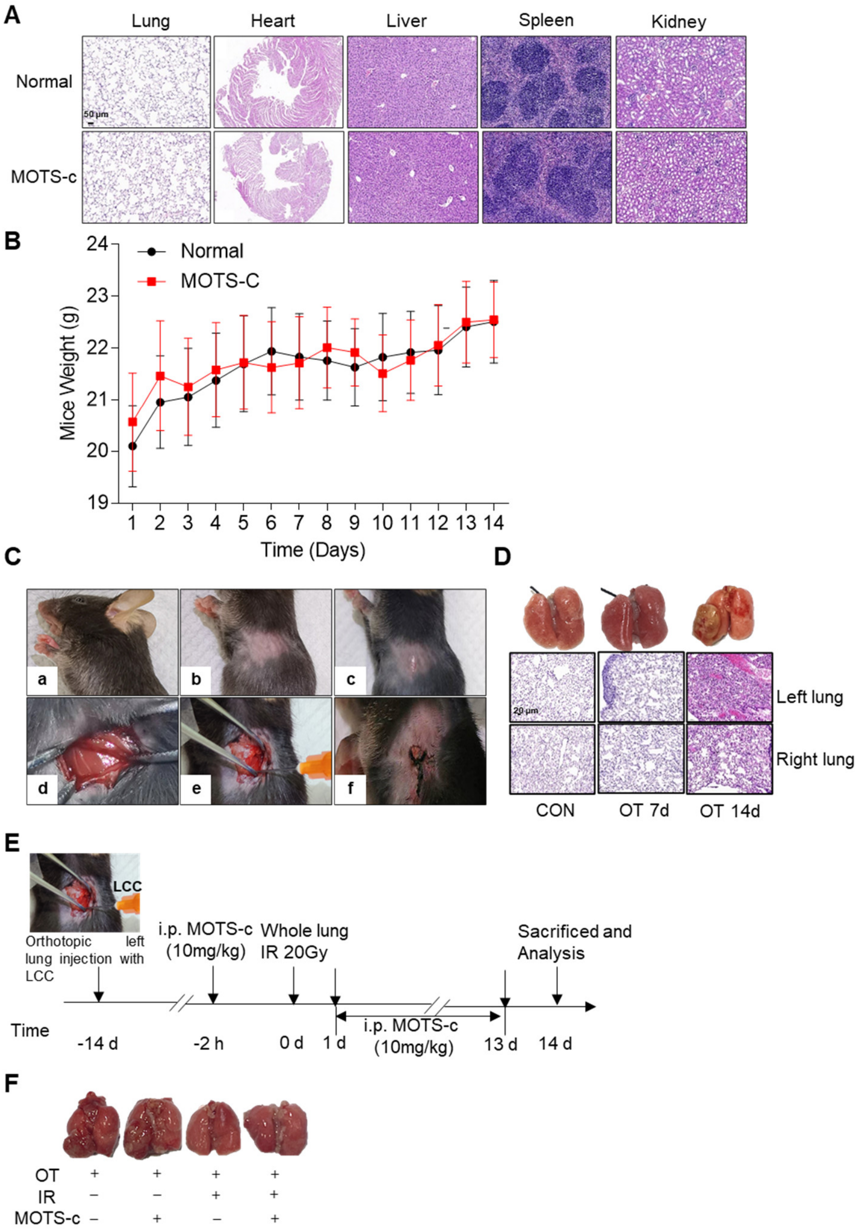

Figure. S1 Effect of MOTS-c on different organs of normal mice and tumor radiotherapy of orthotopic transplantation of lung cancer mice. (A-B) Mice were received intraperitoneal injections of 10 mg/kg MOTS-c peptide dissolved in ddH2O daily for 14 days. (A) Representative images of H&E staining of lung, heart, liver, spleen, and kidney, scale bar, 50 μm, *n*=5. (B) Mice's body weight was measured during MOTS-c intervention for 2 weeks, *n*=5. (C-F) Effect of MOTS-c on the efficacy of radiotherapy in an orthotopic transplantation model of lung cancer. (C) Establishment of orthotopic transplantable model of lung cancer in C57BL/6 mice. (a Anesthesia; b Left lung localization; c Skin preparation; d The incision was opened with forceps to expose the left lung; e Tumor cells were inoculated; f Sutured the incision.) (D) Tumor formation in orthotopic transplantation model of lung cancer. Representative images of H&E staining of lung, scale bar, 20 μm, *n*=5. (E) The schematic timeline of radiation therapy in orthotopic transplantation models of lung cancer. (F) Gross morphology of lung tissue after radiotherapy.

Figure S2

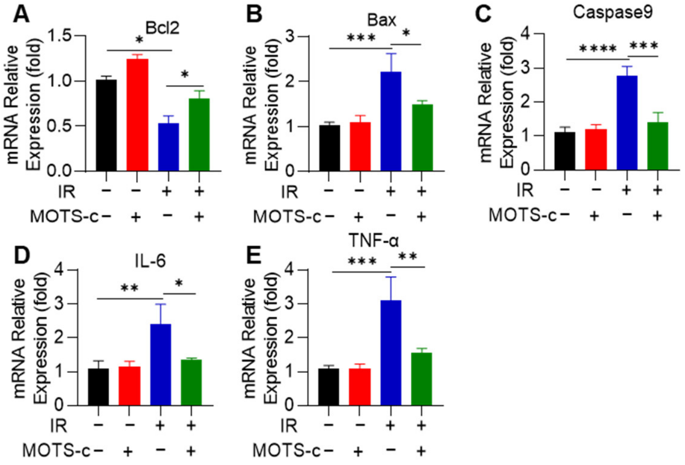

Figure. S2 MOTS-c prevented the apoptosis of alveolar epithelial cells in RP mice. (A-E) The mRNA level of *Bcl2*, *Bax*, *Caspase9*, *IL-6*, and *TNF-α* were measured by quantitative real-time PCR in primary mouse alveolar epithelial cells. *n*=3, **P*<0.05, ***P*<0.01, ****P*<0.001, *****P*<0.0001.

Figure S3

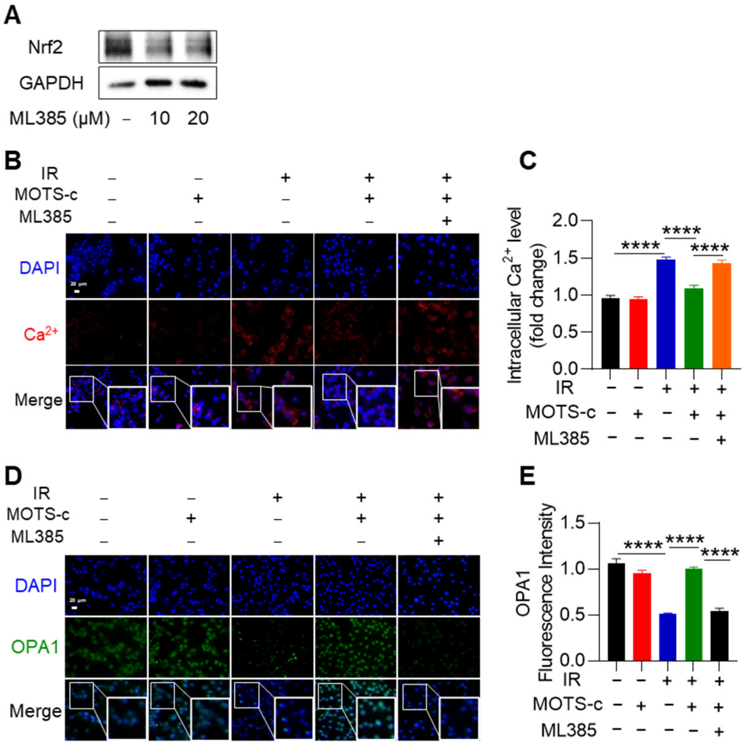

Figure. S3 MOTS-c relieved radiation-induced mitochondrial damage in MLE-12 cells. (A) The expression of GAPDH and Nrf2 were analyzed by western blot. (B, C) Rhod-2, AM Red fluorescent probe (4 μM) was used to detect calcium content in MLE-12, scale bar=20 μm. (D, E) Lung tissue

sections were stained with the OPA1 (green), and DAPI (blue) for immunofluorescence analysis, scale bar=20 µm. *n*=5, ****P<0.0001.

Figure S4

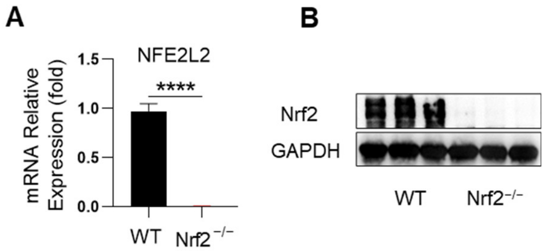

Figure. S4 Gene identification results in WT and Nrf2$^{-/-}$ mice. (A) RT-PCR analysis of *NFE2L2* in WT and Nrf2$^{-/-}$ mice. (B) The expression of GAPDH and Nrf2 were analyzed by western blot. *n*=5, ****P<0.0001.

Figure S5

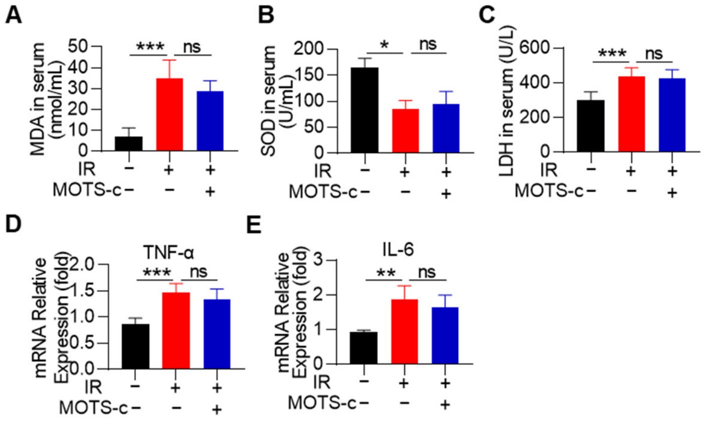

Figure. S5 Nrf2 deficiency abolished the protective function of MOTS-c on RP mice. (A-C) MDA, SOD, and LDH in serum were determined. (D-E) The mRNA levels of IL-6, and TNF-α were measured by quantitative real-time PCR. *n*=5, ns means no significant difference, *P<0.05, **P<0.01, ***P<0.001.
