# Supplementary material for: The Mitochondrial-Derived Peptide MOTS-c Alleviates Radiation Pneumonitis via an Nrf2-Dependent Mechanism
Source: Antioxidants (Basel). 2024 May 17;13(5):613. doi: 10.3390/antiox13050613 (PMC11117534; doi:10.3390/antiox13050613)
Supplement: Supplementary file 1 [file antioxidants-13-00613-s001.zip › Supplementary Table 1.pdf]

Table 1 Primer sequences

Target gene	Primer	Primer sequences (5′–3′)
GAPDH	Forward	TGGCCTTCCGTGTTCCTAC
	Reverse	GAGTTGCTGTTGAAGTCGCA
NFE2L2	Forward	TCTTGGAGTAAGTCGAGAAGTGT
	Reverse	GTTGAAACTGAGCGAAAAAGGC
HO-1	Forward	AAGCCGAGAATGCTGAGTTCA
	Reverse	GCCGTGTAGATATGGTACAAGGA
NQO-1	Forward	AGGATGGGAGGTACTCGAATC
	Reverse	TGCTAGAGATGACTCGGAAGG
Bax	Forward	AGACAGGGGCCTTTTTGCTAC
	Reverse	AATTCGCCGGAGACACTCG
Bcl2	Forward	GAGAGCGTCAACAGGGAGATG
	Reverse	CCAGCCTCCGTTATCCTGGA
Caspase9	Forward	GGCTGTTAAACCCCTAGACCA
	Reverse	TGACGGGTCCAGCTTCACTA
IL-6	Forward	ACTTCCATCCAGTTGCCTTCTTGG
	Reverse	TTAAGCCTCCGATTGTGAAGTG
TNF-α	Forward	CCTGTAGCCCACGTCGTAG
	Reverse	GGGAGTAGACAAGGTACAACCC
SP-C	Forward	ATGGAGAGTCCACCGGATTAC
	Reverse	ACCACGATGAGAAGGCGTTTG
COX I	Forward	GAAGAGACAGTGTTTCATGTGGTGT
	Reverse	TCCTGGGCCTTTCAGGAATA
COX IV	Forward	ATTGGCAAGAGAGCCATTTCTAC
	Reverse	TGGGGAAAGCATAGTCTTCACT
