# Supplementary figures and images for: The Mitochondrial-Derived Peptide MOTS-c Alleviates Radiation Pneumonitis via an Nrf2-Dependent Mechanism
Source: Antioxidants (Basel). 2024 May 17;13(5):613. doi: 10.3390/antiox13050613 (PMC11117534; doi:10.3390/antiox13050613)

### Supplemental Figure.3A

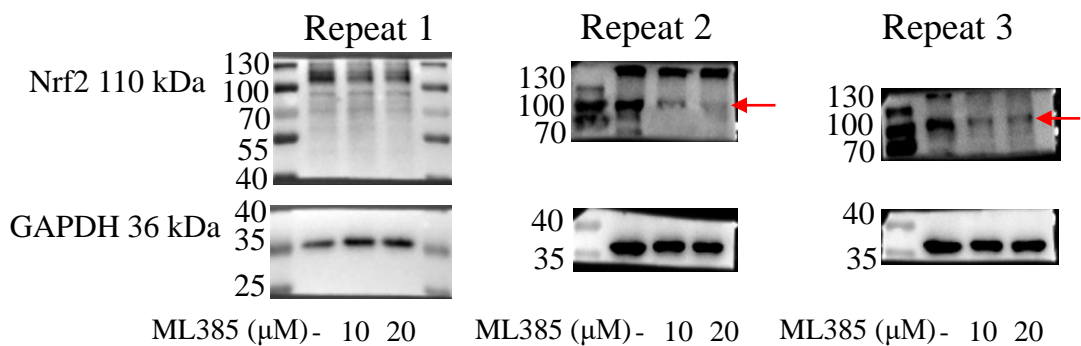

### Supplemental Figure.4B

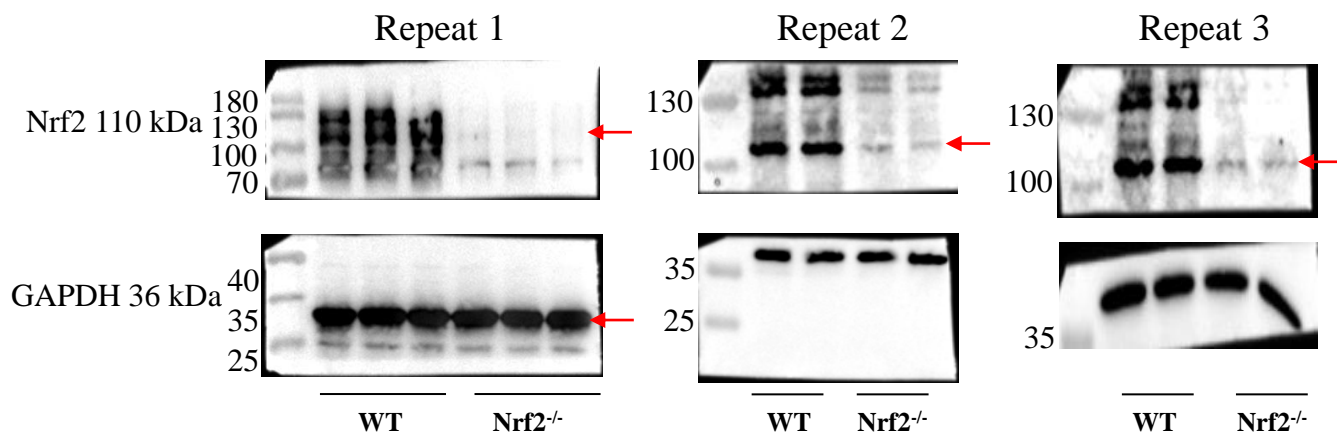

Supplement: Supplementary file 1 [file antioxidants-13-00613-s001.zip › Original images-Supplemental Figures.pdf]
